# Temporal Trend of Age at Diagnosis in Hypertrophic Cardiomyopathy: An Analysis of the International Sarcomeric Human Cardiomyopathy Registry

**DOI:** 10.1161/CIRCHEARTFAILURE.120.007230

**Published:** 2020-09-08

**Authors:** Marco Canepa, Carlo Fumagalli, Giacomo Tini, Justin Vincent-Tompkins, Sharlene M. Day, Euan A. Ashley, Francesco Mazzarotto, James S. Ware, Michelle Michels, Daniel Jacoby, Carolyn Y. Ho, Iacopo Olivotto

**Affiliations:** 1 Cardiovascular Disease Unit, IRCCS Ospedale Policlinico San Martino – IRCCS Italian Cardiovascular Network & Department of Internal Medicine, University of Genova, Italy (M.C., G.T.).; 2 Cardiomyopathy Unit and Genetic Unit, Careggi University Hospital, Florence, Italy (C.F., F.M., I.O.).; 3 MyoKardia Inc, South San Francisco, CA (J.V.-T.).; 4 Department of Internal Medicine, University of Michigan, Ann Arbor (S.M.D.).; 5 Stanford Center for Inherited Heart Disease, CA (E.A.A.).; 6 National Heart and Lung Institute and National Institute for Health Research Royal Brompton Cardiovascular Biomedical Research Unit, Imperial College London, United Kingdom (F.M., J.S.W.).; 7 Department of Cardiology, Thoraxcenter, Erasmus Medical Center Rotterdam, the Netherlands (M.M.).; 8 Yale University, New Haven, CT (D.J.).; 9 Cardiovascular Division, Brigham and Women’s Hospital, Boston, MA (C.Y.H.).

**Keywords:** cardiomyopathy, hypertrophic, genotype, heart failure, phenotype, prevalence

## Abstract

**Background:**

Over the last 50 years, the epidemiology of hypertrophic cardiomyopathy (HCM) has changed because of increased awareness and availability of advanced diagnostic tools. We aim to describe the temporal trends in age, sex, and clinical characteristics at HCM diagnosis over >4 decades.

**Methods:**

We retrospectively analyzed records from the ongoing multinational Sarcomeric Human Cardiomyopathy Registry. Overall, 7286 patients with HCM diagnosed at an age ≥18 years between 1961 and 2019 were included in the analysis and divided into 3 eras of diagnosis (<2000, 2000–2010, >2010).

**Results:**

Age at diagnosis increased markedly over time (40±14 versus 47±15 versus 51±16 years, *P*<0.001), both in US and non-US sites, with a stable male-to-female ratio of about 3:2. Frequency of familial HCM declined over time (38.8% versus 34.3% versus 32.7%, *P*<0.001), as well as heart failure symptoms at presentation (New York Heart Association III/IV: 18.1% versus 15.8% versus 12.6%, *P*<0.001). Left ventricular hypertrophy became less marked over time (maximum wall thickness: 20±6 versus 18±5 versus 17±5 mm, *P*<0.001), while prevalence of obstructive HCM was greater in recent cohorts (peak gradient >30 mm Hg: 31.9% versus 39.3% versus 39.0%, *P*=0.001). Consistent with decreasing phenotypic severity, yield of pathogenic/likely pathogenic variants at genetic testing decreased over time (57.7% versus 45.6% versus 38.4%, *P*<0.001).

**Conclusions:**

Evolving HCM populations include progressively greater representation of older patients with sporadic disease, mild phenotypes, and genotype-negative status. Such trend suggests a prominent role of imaging over genetic testing in promoting HCM diagnoses and urges efforts to understand genotype-negative disease eluding the classic monogenic paradigm.

**What is New?**

- In this analysis from a large international registry, we observed how patients with hypertrophic cardiomyopathy, irrespective of the geographic region of origin, have become older at presentation in recent cohorts, often asymptomatic, with milder phenotypes and a more frequently negative or inconclusive genetic test.

**What are the Clinical Implications?**

- Our findings likely reflect a greater physician awareness and diagnostic sensitivity in the medical community. The increasing number of hypertrophic cardiomyopathy diagnoses in older patients with milder phenotype and sporadic disease will significantly change the therapeutic and prognostic landscape of this condition and further questions its classic monogenic paradigm.

Once considered a rare disease of the young, hypertrophic cardiomyopathy (HCM) is now recognized as relatively common and increasingly diagnosed in middle-aged and older adults,^1,2^ burdened by greater risk of developing atrial fibrillation and heart failure than sudden cardiac death.^3,4^ Older age at diagnosis in recent years pairs with the global aging of populations but also with greater HCM disease awareness and widespread use of cardiac imaging, particularly echocardiography. Both have resulted in increasing rates of incidental diagnoses in otherwise asymptomatic individuals.^5,6^ Advances in genetic testing may also have played a role, mostly by virtue of cascade family screening.^7^

A comprehensive perception of this trend and its implications based on large multinational HCM populations may help shape future diagnostic and prognostic algorithms and allocate clinical and genetic resources.

We herein describe the temporal trends in age, sex, and clinical characteristics at HCM diagnosis in patients enrolled in the international Sarcomeric Human Cardiomyopathy Registry (SHaRe) over the last 4 decades.

## Methods

The SHaRe registry is an international database created by 11 HCM centers, which comprises over 7000 patients.^4^ The registry conforms to the principles of the Helsinki declaration and the local institutional review boards approved the study protocol. All participants gave informed consents. The data will not be made available to other researchers for purposes of reproducing the results or replicating the procedure because of constraints related to human subjects

research. Analytical methods will be made available on request. For this analysis, records were updated to the first quarter of 2019. We retrospectively reviewed clinical records of all SHaRe HCM patients diagnosed at an age ≥18 years. Temporal trends in parameters of interest were plotted by quinquennial periods. In addition, clinical and instrumental data at diagnosis were stratified into 3 groups: patients diagnosed <2000, 2000 to 2010, and >2010.

Age at HCM diagnosis was distinguished from age at initial site evaluation. HCM was defined by the presence of increased asymmetrical left ventricular (LV) wall thickness ≥13 mm in the absence of abnormal loading conditions.^4^ Genetic testing was performed at all sites using the platforms locally available over time. Variants in the sarcomeric genes were classified as pathogenic or likely pathogenic (SARC+), variant of unknown significance (SARC VUS), or no pathogenic variants (SARC−) by each site using contemporary criteria and through a subsequent standardization.^4^

### Statistical Analysis

Data are expressed as percentages, mean and SD, or median with interquartile range for skewed distributions. Temporal trends in the main characteristics of patients at enrollment in the registry were analyzed using parametric tests (Student *t* test, or ANOVA when >2 input variable categories were present) and nonparametric when necessary (for non-normal distributions—Mann-Whitney *U* test or Kruskal-Wallis test when >2 input variable categories were present) for continuous variables and the χ^2^ or Fisher exact test (if cell count was <5 in one of the cells) for categorical data. Statistical analysis was performed with SPSS v24.0 (IBM, Armonk, NY).

## Results

Overall, 7286 patients with HCM, diagnosed between 1961 and March 2019 at 6 US (n=3212) and 5 non-US (n=4074) participating centers, were included in the analysis (Table). The number of HCM diagnoses was low before 2000 and increased significantly after 2000 (35/y <2000 versus 272/y in 2000–2010 versus 357/y >2010), particularly in US sites. Patients were progressively older at HCM diagnosis (40±14 versus 47±15 versus 51±16 years in patients diagnosed <2000 versus 2000–2010 versus >2010 respectively, *P*<0.001), with a similar trend in US and non-US sites (Table, Figure [A]). Rate of diagnoses >60 years increased from 9.2% before 2000 to 31.8% after 2010. Notably, prevalence of patients diagnosed at >70 years reached 10.7% after 2010 (Table). Male-to-female ratio remained stable at about 3:2 (Figure [B]). Starting in 1984, women were significantly older than men at diagnosis, with a mean age gap of 4.5±0.6 years (Figure [B]). The number of diagnoses in nonwhite individuals increased over time, as well as the prevalence of hypertension and obesity (Table). Most patients were first family member presenting for care at the site (ie, probands), without significant changes over time (Table).

**Table.**
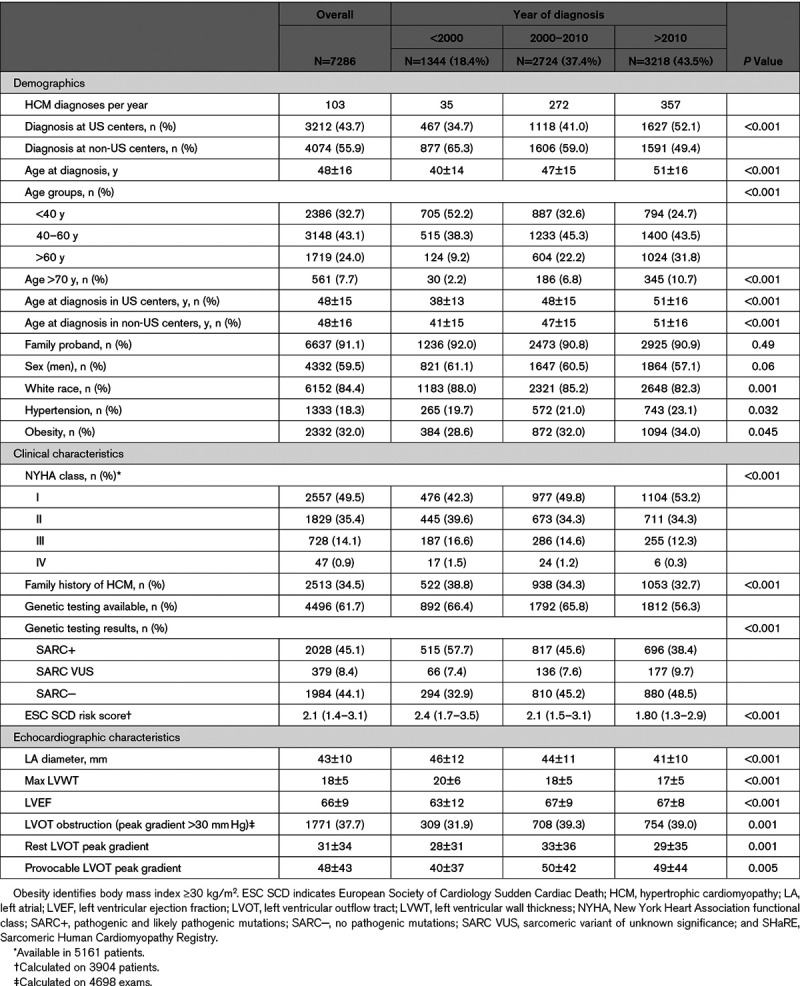
Baseline Characteristics of Patients Diagnosed With HCM in the SHaRE Registry Overall and by Year of Diagnosis

**Figure.**
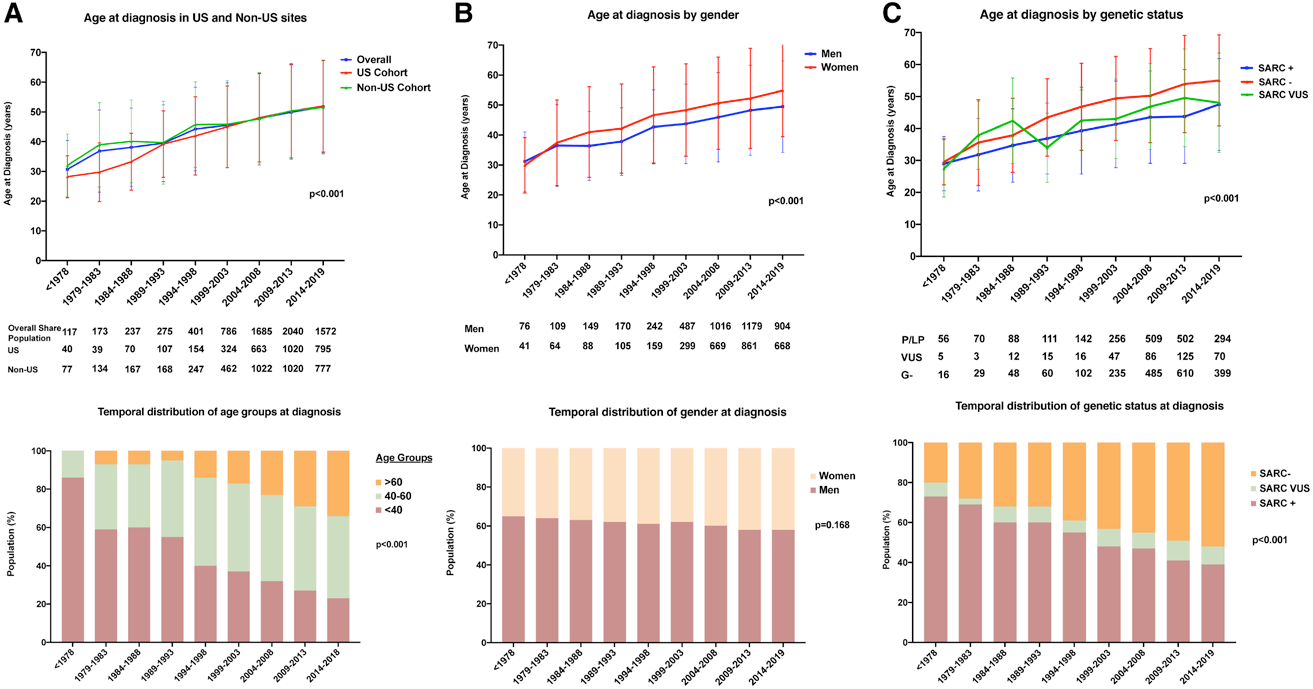
**Temporal trends and distributions in age at hypertrophic cardiomyopathy diagnosis by site, sex, and genetic status.** SARC+ indicates pathogenic and likely pathogenic mutations; SARC−, no pathogenic mutations; and SARC VUS, sarcomeric variant of unknown significance.

Prevalence of patients with positive HCM family history declined during the study period (38.8% versus 34.3% versus 32.7%, *P*<0.001). Genetic testing was performed in 4496 patients (61.7% of the overall population): SARC+ variants were identified in 2028 (45.1%; Table). SARC− patients were diagnosed at an older age than those with SARC VUS or SARC+ throughout the whole study period (Figure [C]). The yield of genetic testing gradually declined with time, from 57.7% SARC+ before 2000 to 38.4% SARC+ after 2010, paralleled by a concomitant increase in SARC VUS (Table; Figure [C]).

Severity of heart failure symptoms at presentation declined after 2000 (New York Heart Association III/IV: 18.1% versus 15.8% versus 12.6%, *P*<0.001), with progressive emergence of asymptomatic patients (Table). Maximal LV wall thickness at diagnosis significantly decreased over time (20±6 versus 18±5 versus 17±5 mm, *P*<0.001), while obstructive HCM was progressively more prevalent (Table). Moreover, patients diagnosed after 2010 had smaller left atrial diameters and higher LV ejection fraction. This collective trend translated in a progressively lower estimated risk of sudden cardiac death at diagnosis, according to the 2014 European Society of Cardiology prediction model (Table).

## Discussion

The number of HCM diagnoses has steadily increased worldwide over the last 40 years, with dramatic change in the perception of the disease and its epidemiology (from rare/malignant to relatively common/relatively favorable). In this analysis from a large international registry, we observed how patients with HCM, irrespective of the geographic region of origin, have become older at presentation in recent cohorts, often asymptomatic, with milder phenotypes and a more frequently negative or inconclusive genetic test. A similar trend has been previously described in an Italian nationwide survey of 1677 patients with HCM conducted in the year 2002.^1^ Specifically, age at HCM diagnosis was found to increase from an average of 36 years before 1982 to 44 years after 1992. Subsequent administrative US data have confirmed that the average age of patients with HCM known today falls in the fifth decade of life.^2^ In the SHaRe registry, mean age at HCM diagnosis after 2010 was 51±16 years, and females were consistently older than male patients. These findings were paralleled by an evolving perception of the disease spectrum, moving from classic to atypical and less dramatic phenotypes. Patients in New York Heart Association functional class I, constituting 42.3% of the total SHaRe cohort before 2000, peaked at >53% after 2010. This is in accordance with the seminal findings from the CARDIA study, where an unexpectedly high prevalence was found by echocardiographic population screening, revealing a majority of asymptomatic, undiagnosed HCM subjects in the community.^8^ The fact that more asymptomatic HCM individuals are identified in the clinical setting (as opposed to population screening initiatives) reflects greater physician awareness and diagnostic sensitivity.^5^

Our findings, combined with prior reports, stimulate a number of relevant epidemiological considerations. First, the contemporary size of known HCM populations are still far from the total number of patients expected based on national estimates of 1:500 to 1:3000 to 3500 individuals.^2^ Even accounting for a considerable proportion of individuals not followed at academic centers—and therefore unreported in the literature—the ultimate, real-world profile of HCM is being progressively uncovered but still not completely unraveled. Second, the exponential increase in HCM diagnoses seems because of the systematic exploitation of electrocardiography and echocardiography, rather than to more advanced diagnostic tools.^9,10^ Indeed, the boom in HCM diagnoses occurred around the year 2000, with some limited increase after that date. Based on this simple temporal criterion, novel technologies such as cardiac magnetic resonance and next generation sequencing genetics thus seems to have contributed poorly to this epidemiological shift.^11^ Third, the number of diagnoses in the classical niche of HCM (young males with marked hypertrophy, familial disease, and SARC+ carriers) has remained substantially stable after the year 2010, and the multiplication in cohort size is increasingly due to the inclusion of older, genotype-negative patients with sporadic disease and less marked LV hypertrophy. The higher prevalence of the obstructive phenotype in recent cohorts seems to counter this general trend; however, LV obstruction has been described as a frequent feature of older, generally SARC−, patients with HCM^12^ and attributed to geometric and functional modifications of the left ventricular outflow tract in relation to age.^3,13^

Overall, characteristics at diagnosis of contemporary HCM cohorts significantly differ from the classic disease described in the 1960s and 1970s. Consistently, we observed a growing number of diagnoses in genotype-negative HCM, as opposed to high gene testing yields in the early cohorts. Recently, there has been a call to arms to address this knowledge gap, and the classic HCM monogenic paradigm has been questioned.^11^ Our results embrace this view and force us to speculate regarding the pathogenic mechanisms on the basis of the increasing cohort of SARC VUS carriers and genotype-negative patients, accounting for about 60% of HCM diagnoses after 2010 in the SHaRe registry population. The availability of more genes and variants in larger next generation sequencing panels has likely contributed to increasing the number of SARC VUS, but not SARC+ patients in later years. This does not question the seminal theory of HCM as a disease of the sarcomere—which has passed the test of time and has proven essential in developing novel, groundbreaking therapies.^14^ However, it is by now clear that beyond a typical HCM phenotype, lay diverse subsets of phenotypes which seems to escape a monogenic logic and involve other, still unknown, mechanisms. The epidemiological picture emerging from the present suggests the need for additional efforts involving clinical and basic science, as well as the opportunity to re-think classic manifestations of disease and their implications for screening strategies, risk stratification, and allocations of resource.

## Conclusions

Rapidly expanding international HCM populations include progressively older patients, with sporadic disease, mild phenotypes, and genotype-negative status. This temporal trend suggests a driving role of imaging over genetic testing in enhancing HCM diagnoses and urges efforts to understand genotype-negative disease eluding the classic monogenic paradigm.

## Sources of Funding

Funding for SHaRe has been provided through an unrestricted research grant from Myokardia, Inc, a startup company that is developing therapeutics that target the sarcomere. MyoKardia, Inc, had no role in approving the content of this manuscript. Dr Day is supported by funding from the National Institutes of Health (R01 GRANT11572784), the American Heart Association (grant in aid), and the Taubman Medical Institute (University of Michigan). Dr Ware is supported by the Wellcome Trust (107469/Z/15/Z) and the Medical Research Council (United Kingdom). Dr Ho is supported by funding from the National Institutes of Health (1P50HL112349 and 1U01HL117006). Dr Olivotto was supported by the European Union’s Horizon 2020 Research and Innovation Programme under Grant Agreement no. 777204: SILICOFCM—In Silico trials for drug tracing the effects of sarcomeric protein mutations leading to familial cardiomyopathy; by the Italian Ministry of Health (left ventricular hypertrophy in aortic valve disease and hypertrophic cardiomyopathy): genetic basis, biophysical correlates, and viral therapy models (RF-2013-02356787), and NET-2011-02347173 (mechanisms and treatment of coronary microvascular dysfunction in patients with genetic or secondary left ventricular hypertrophy) and by the Ente Cassa di Risparmio di Firenze (bando 2016) juvenile sudden cardiac death: just know and treat.

## Disclosures

Drs Day, Ho, Olivotto, and Ashley receive research support from Myokardia, Inc. The other authors report no conflicts related to the present work.
